# Investigation of *PtSGT1* and *PtSGT4* Function in Cellulose Biosynthesis in *Populus tomentosa* Using CRISPR/Cas9 Technology

**DOI:** 10.3390/ijms222413200

**Published:** 2021-12-07

**Authors:** Yinxuan Xue, Siyan Li, Deyu Miao, Sai Huang, Bin Guo, Shanwen Li, Xin-Min An

**Affiliations:** 1 Beijing Advanced Innovation Center for Tree Breeding by Molecular Design, National Engineering Laboratory for Tree Breeding, Key Laboratory of Genetics and Breeding in Forest Trees and Ornamental Plants, Ministry of Education, College of Biological Sciences and Technology, Beijing Forestry University, Beijing 100083, China; xueyinxuan@bjfu.edu.cn (Y.X.); kenneth1993@bjfu.edu.cn (S.L.); miaodeyu@163.com (D.M.); HUANGSai@bjfu.edu.cn (S.H.); guobin531188058@163.com (B.G.); 2 Institute of Forest Genetics and Tree Breeding, Shanxi Academy of Forestry and Grassland Sciences, Taiyuan 030012, China; 3 Institute of Forest Culture, Shandong Academy of Forestry, Jinan 250014, China; lishanwen66@163.com

**Keywords:** sitosterol glycosyltransferase, *Populus tomentosa*, gene editing, cellulose synthesis, cell wall

## Abstract

Cellulose synthesis is a complex process in plant cells that is important for wood processing, pulping, and papermaking. Cellulose synthesis begins with the glycosylation of sitosterol by sitosterol glycosyltransferase (SGT) to produce sitosterol-glucoside (SG), which acts as the guiding primer for cellulose production. However, the biological functions of SGTs in *Populus tomentosa* *(P. tomentosa)* remain largely unknown. Two full-length *PtSGT* genes (*PtSGT1* and *PtSGT4*) were previously isolated from *P. tomentosa* and characterized. In the present study, CRISPR/Cas9 gene-editing technology was used to construct *PtSGT1-*sgRNA and *PtSGT4-*sgRNA expression vectors, which were genetically transformed into *P. tomentosa* using the *Agrobacterium*-mediated method to obtain transgenic lines. Nucleic acid and amino acid sequencing analysis revealed both base insertions and deletions, in addition to reading frame shifts and early termination of translation in the transgenic lines. Sugar metabolism analysis indicated that sucrose and fructose were significantly downregulated in stems and leaves of mutant *PtSGT1-*1 and *PtSGT4-*1. Glucose levels did not change significantly in roots and stems of *PtSGT1-*1 mutants; however, glucose was significantly upregulated in stems and downregulated in leaves of the *PtSGT4-*1 mutants. Dissection of the plants revealed disordered and loosely arranged xylem cells in the *PtSGT4-*1 mutant, which were larger and thinner than those of the wild-type. This work will enhance our understanding of cellulose synthesis in the cell walls of woody plants.

## 1. Introduction

Cell walls are important components of plant cells responsible for their characteristic structures. In addition to protecting and supporting plant cells, cell walls contribute to their physiological functions, such as material transport and signal transduction. Cellulose is the most important component of the plant cell wall. The basic unit of cellulose is pyranoid D-glucose, which is linked by β-1,4-glycosidic bonds. In plants, sterol biosynthesis occurs through the mevalonate and non-mevalonate pathways [1], which have highly diverse skeletal and glycosylated structural forms. Sterols and their derivatives are involved in the structure of cell membranes [2], and act as precursors for hormone and vitamin biosynthesis [3]. Other important cellular functions of sterols include anti-stress and anti-heat shock actions, signal transduction, and protection of bacteria from the host immune response [4]. Some sterols, such as sitosterol, are ubiquitous in plants [5].

Glycosyltransferases are a multigene family of enzymes that catalyze the glycosylation of biomolecules; there are 98 families distinguished according to amino acid sequence similarity [6]. Sitosterol glycosyltransferase (SGT) catalyzes the glycosylation of phytosterols and is associated with membrane binding [7]. Sitosterol-glucoside (SG) is derived from sterol, a component of the plant cell membrane [8], and is used as a primer for cellulose synthesis [9]. Cellulose synthase (CesA) utilizes uridine 5′-diphosphoglucose (UDP-Glu) as a substrate to form glucan chains as β-1,4-glycosidic bonds [9]. Peng [10] demonstrated that cotton fiber membranes synthesize sitosterol-β-glucoside (SG), while UDP-Glu synthesized sitosterol-cellulose dextrin (SCD) in the presence of pre-synthesized cellulose. As a primer, SG participated in the initial cellulose synthesis. Sucrose is one of the photosynthetic products transported from source to sink in plants [11]. Sucrose synthase (SUS, EC 2.4.1.13) is the major sucrolytic enzyme [12]. It catalyzes a reversible reaction, but preferentially converts sucrose into fructose and UDP-Glu *in planta* [12], while as the substrate of glucan chains polyreaction, UDP-Glu is involved in cellulose synthesis in the cell walls of woody plants.

The proposed function of the Korrigan (Kor) protein is cleavage of SG from SCD, which allows the chain to be more efficiently extended. Studies have shown that cellulose biosynthesis occurs in herbicide-treated cotton fibers co-transformed with SG and cellulose fragments, indicating the importance of these biomolecules as primers for cellulose biosynthesis in higher plants [10]. Schrick et al. [13] reported that the phytosterol biosynthetic mutants *fackel*, *hydra1*, and phytosterol methyltransferase reduced cellulose levels. Using UDP-Glu and β-sitosterol as substrates, Li et al. [14] found that GhSGT1 and GhSGT2 recombinant proteins in cotton displayed different SG enzyme activities. The biochemical properties of *GhSGT2* are similar to those of *GhCESAs*, and *GhSGT2* may play an important role in the cellulose biosynthesis of cotton fibers. Many reports on cellulose synthesis exist, with most being focused on the role of the *SGT* gene in cellulose synthesis in herbaceous plants, such as *Arabidopsis thaliana* [15] and cotton [14]; however, there are few studies on woody plants. Studying the *SGT* gene in *P. tomentosa* could provide a foundation for further analysis of cellulose synthesis in woody plants.

Poplar is a fast-growing tree with high economic and ecological value [16,17]. In 2006, the entire genome of *Populus trichocarpa (P. trichocarpa)* was published [18]. Many researchers have identified the poplar tree as a model system for tree biology due to its small genome, ease of cloning, and amenability to high-throughput transgenic techniques [19]. Liu et al. [20] analyzed the efficiency of mutagenesis by using single-guide RNA (sgRNA) targeting the phytoene desaturase (*PtoPDS*) DNA sequence of *P. tomentosa*. A mismatch was found between sgRNA and the target gene, leading to reduced efficiency of mutagenesis, and possible mutagenesis failure. Complementarity of sequences between the 3′-terminal nucleotides of sgRNA and target gene was found to be particularly important for effective mutagenesis. In another similar study, the method utilized CRISPR/Cas9 technology to destroy endogenous *PtoPDS* genes while also producing homologous and heterologous PDS mutants, indicating that the CRISPR/Cas9 system is an efficient and powerful tool for genomic modification of woody plants [21].

CRISPR/Cas9 technology was also used to knock out 4CL genes in poplar trees; 100% mutagenesis was achieved in two 4CL genes, supporting the specificity and effectiveness of the CRISPR/Cas9 system in these plants [22]. This provided a simple way to identify the functions of members of a polygenic family. Studies have shown that CRISPR/Cas9 technology effectively produces targeted mutations in transgenic poplars, and homozygous mutations are produced at the desired site in the first generation [21]. At present, most research on CRISPR/Cas9 technology focuses on model plants and crops, with few studies of woody plants. Effective mutation of woody plants by the CRISPR/Cas9 system will be beneficial by allowing for genetic enhancement of forest trees. In our previous preliminary analysis, four *PtSGT*s of eight total members were isolated from *P. tomentosa* and characterized (Supplementary Figure S1 and Supplementary Table S2). Combining with the characteristics of high expression of *AtSGT1* and *AtSGT4* in stems and leaves of *Arabidopsis* based on TAIR (TAIR—Home Page arabidopsis.org) database, of these, *PtSGT1* and *PtSGT4* were chosen for further functional identification due to their high expression in stems and leaves. In this study, the *P. tomentosa PtSGT1* and *PtSGT4* genes were mutated using CRISPR/Cas9, which altered the sugar content and xylem structure in the mutant lines. Our findings will further our understanding of cellulose biosynthesis in the cell walls of woody plants.

## 2. Results

### 2.1. Cloning and Sequence Analysis of PtSGT1 and PtSGT4

**Figure 1:**
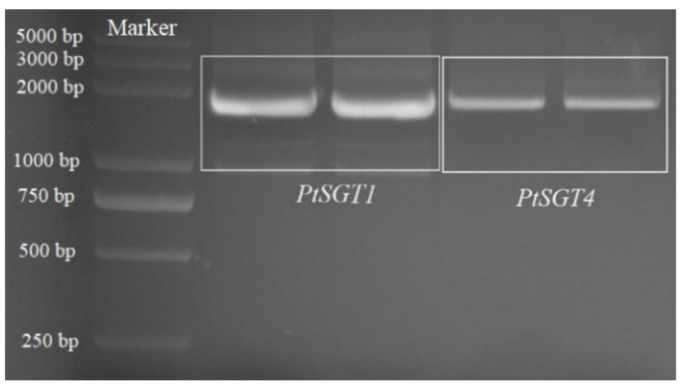
Electropherograms of *PtSGT1* and *PtSGT4* gene clones.

Following amplification with primers PtSGT1F, PtSGT1R, PtSGT4F, and PtSGT4R, a 1.9-kb fragment was obtained by 1.5% agarose gel electrophoresis using *P. tomentosa* cDNA as the template (Figure 1). The sequencing results indicate that the full-length cDNAs of *P. tomentosa PtSGT1* (GenBank acc. no. MG904686) and *PtSGT4* (unpublished) are 1851 and 1935 bp, respectively, and the CDS sequences of both are complete open reading frames (ORF) encoding 616 and 644 amino acids, respectively.

TargetP 1.1 Server software was used to predict the subcellular localization of the two genes. *PtSGT1* was thought to be located in the chloroplast, while *PtSGT4* may be in the cytoplasm. Blast was used to analyze the nucleic acid sequence similarity of *Populus* *trichocarpa*, and indicated 94% similarity between *PtSGT1* and *PtrSGT1* (Potri.014G178300.1)*,* and 99% similarity between *PtSGT4* and *PtrSGT4* (Potri.005G193100.1). The results of the BLASTP (protein–protein blast) comparison indicated that the amino acid sequences encoded by *P. tomentosa PtSGT1* and *PtSGT4* were 83% and 96% similar to those of *P. trichocarpa*, respectively.

### 2.2. Plasmid Construction

**Figure 2:**
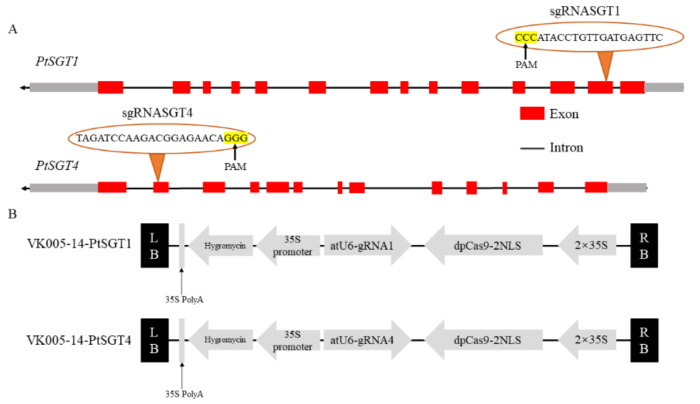
Design of the sgRNAs for the target genes and construction of the CRISPR/Cas9-sgRNA structure: (**A**) Target sequence selection site for the *PtSGT* genes. Untranslated regions (UTRs) are shown in gray boxes. (**B**) Schematic illustration of the CRISPR/Cas9-sgRNA structure (the backbone is VK005-14, dpCas9: SpCas9 optimized for dicotyledons, NLS: nuclear localization sequence).

Table 1 shows the sgRNAs designed for *PtSGT1* and *PtSGT4*, respectively, which were integrated with the plasmid vector to obtain the recombinant expression vector VK005-14 (Figure 2B). The positions of the sgRNAs in the genes are shown in Figure 2A. After being transferred into *E. coli* DH5α cells, single-colony PCR detection was carried out, and positive *E. coli* colonies were collected for plasmid extraction. The tested vectors were introduced into *Agrobacterium* for verification by PCR. The PCR product was subjected to agarose electrophoresis, and positive clones were selected. The detected fragment size was consistent with the size of the target gene, indicating that the recombinant vector extracted from *E. coli* had been successfully transferred into *Agrobacterium*.

### 2.3. Genetic Transformation of P. tomentosa

**Figure 3:**
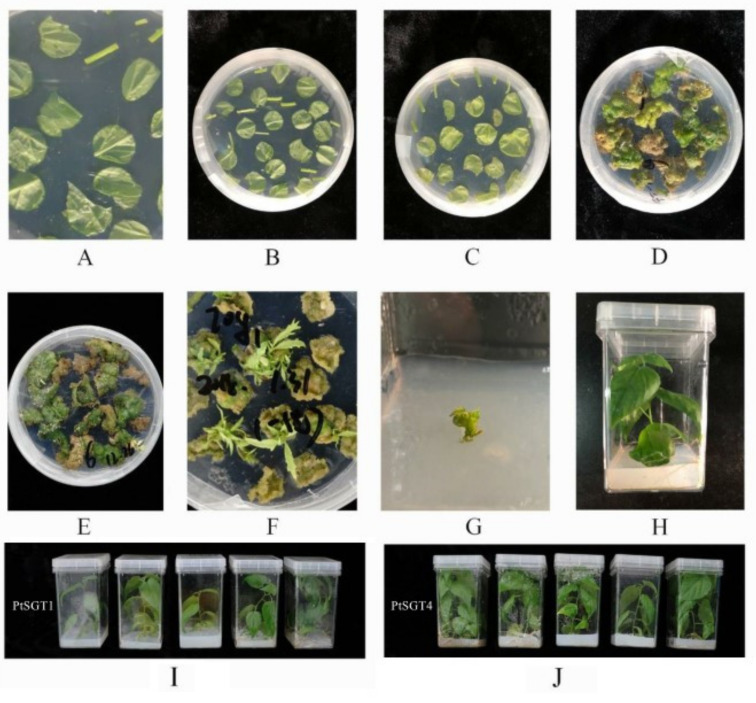
*Agrobacterium*-mediated genetic transformation of *P*. *tomentosa:* (**A**) plant tissue preculture (Murashige and Skoog (MS) medium with 2 mg/L 6-benzylaminopurine (6-BA) and 0.1 mg/L 1-naphthylacetic acid (NAA)). (**B**,**C**) Culture after *Agrobacterium* infection (MS medium with 2 mg/L 6-BA and 0.1 mg/L NAA and 500 mg/L cefotaxime (Cef) and 1 mg/L hygromycin (Hyg)). (**D**–**F**) Adventitious bud growth (MS medium with 2 mg/L 6-BA and 0.1 mg/L NAA and 500 mg/L Cef and 1 mg/L Hyg). (**G**) Adventitious buds were transplanted into rooting medium (1/2 MS medium with 0.4 mg/L indole-3-butytric acid (IBA) and 500 mg/L Cef and 1 mg/L Hyg). (**H**) Adventitious buds developed into plants (1/2 MS medium with 0.4 mg/L IBA and 500 mg/L Cef and 1 mg/L Hyg). (**I**,**J**) Plants to be tested (1/2 MS medium with 0.4 mg/L IBA).

**Figure 4:**
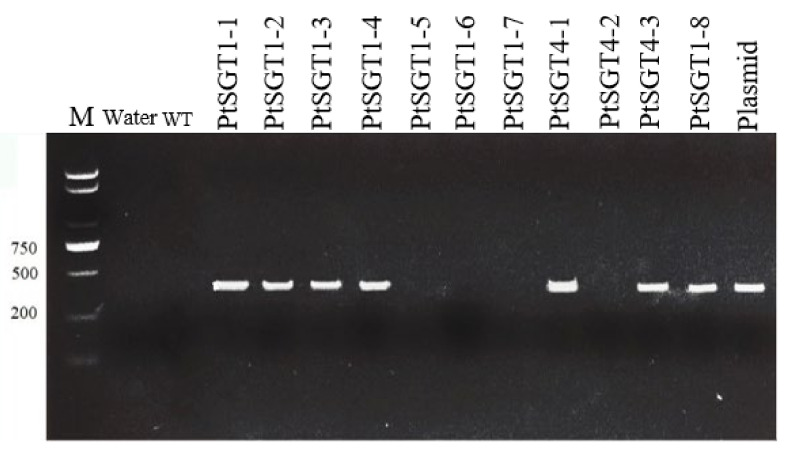
Part of the electrophoretic detection map of transgenic seedlings: M, marker (100–2000 bp, Sangon Biotech, Shanghai, China); Water, negative control; WT, wild-type; Plasmid, positive control. PtSGT1-1, 1-2, 1-3, 1-4, 1-6, 1-7, 1-8, and PtSGT4-1, 4-2, 4-3 represent different transformants, respectively.

Transgenic plants were obtained by transforming vector ‘p2×35S-dpCas9-atU6-gRNA-35sh-Hyg′ into *Agrobacterium tumefaciens* (Figure 3), and DNA from the leaf of 30-day-grown mutant was extracted. This extracted DNA was used as the template and the universal detection primer was used for detection (Table 2). A total of 51 strains of positive plants were obtained and WT *P. tomentosa* was used as the negative control (Figure 4).

**Figure 5:**
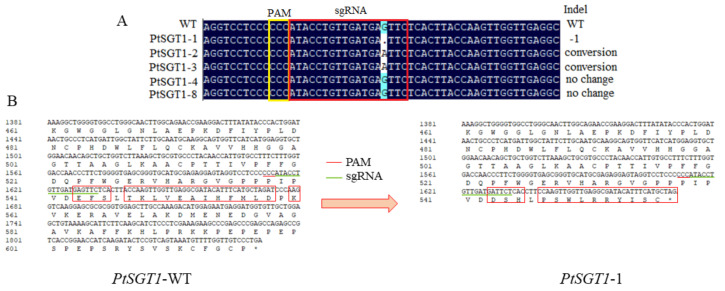
Mutation analysis of the target sequences in transgenic lines of *P. tomentosa* with edited *PtSGT1*: (**A**) results of the target sequencing, (**B**) comparison of the amino acid sequences of wild-type and a transgenic line with edited *PtSGT1*.

**Figure 6:**
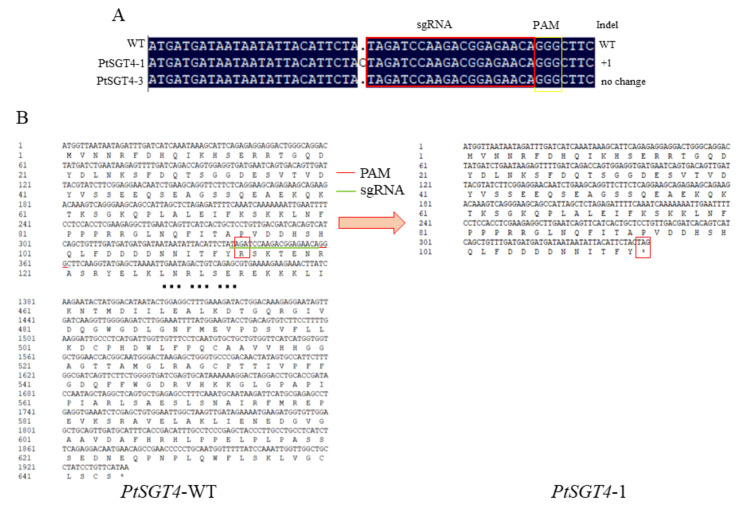
Mutation analysis of the target sequences in transgenic lines of *P. tomentosa* with edited *PtSGT4*: (**A**) results of target sequencing. (**B**) Comparison of the amino acid sequences of wild-type and a transgenic line with edited *PtSGT4*.

Comparisons of WT and positive mutant sequences identified two target positive mutants of *PtSGT1* (Figure 5) and *PtSGT4* (Figure 6) with sequence changes. *PtSGT1*-2 and *PtSGT1*-3 had single base conversions, with the original G base changed to an A base, and a deletion of the G base at the same position of *PtSGT1*-1. The sequence of *PtSGT4*-1 had a single base increase. The amino acid of the WT *PtSGT4* gene and transgenic types *PtSGT1*-1 and *PtSGT4*-1 products were compared using DNAMAN software, which identified large amino acid changes at the end of the *PtSGT1* sequence (Figure 5). Few changes to the amino acid sequence of *PtSGT4* were observed; however, the single base change in both resulted in the premature end of translation (Figure 6).

### 2.4. Sugar Extraction and Measurement

**Figure 7:**
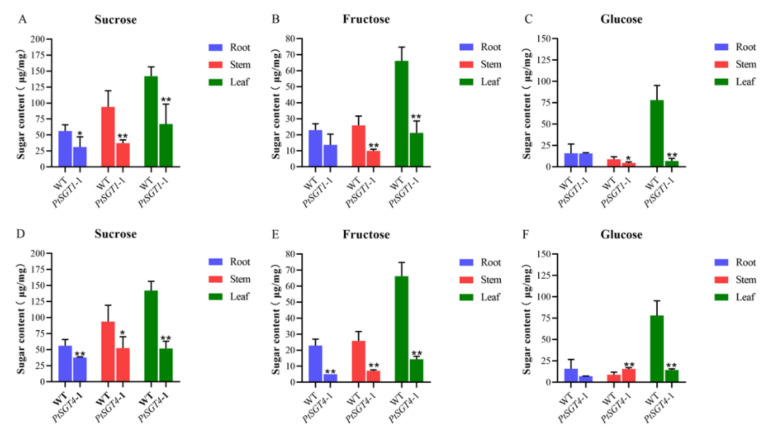
Comparison of sugar content between wild-type and transgenic type: (**A**–**C**) comparison of the sucrose, fructose, and glucose levels between the wild-type and transgenic *PtSGT1*-1; (**D**–**F**) comparison of the sucrose, fructose, and glucose levels between the wild-type and transgenic *PtSGT4*-1.

Figure 7 shows the content of sucrose, fructose, and glucose in *PtSGT1*-1 and *PtSGT4*-1 transgenic plants. Sucrose and fructose levels in roots, stems, and leaves of the *PtSGT1*-1 mutant were significantly lower than those of WT *P. tomentosa* and gene-edited *P. tomentosa*, with the levels being reduced by 40–70%. The glucose content in leaves of transgenic plants was significantly lower than in WT. Sucrose and fructose levels in roots, stems, and leaves of the *PtSGT4*-1 mutant were significantly lower than those of WT, while the glucose content in the stem was higher than that in WT.

### 2.5. Sections of Xylem from Transgenic Plants

**Figure 8:**
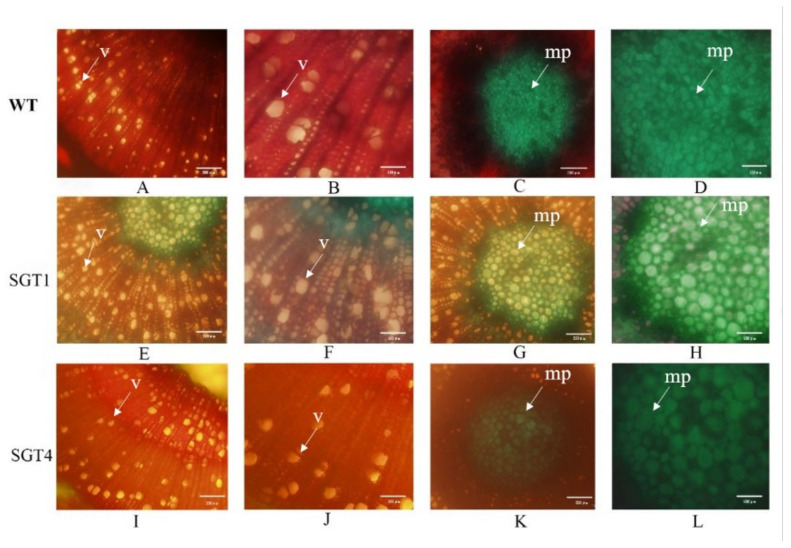
Xylem sections from wild-type and transgenic *PtSGT1*-1 and *PtSGT4*-1 plants. The arrows point to the vessel (v) and myeloid parenchyma (mp). Images (**A**–**D**) are 10 × and 20 × magnifications of xylem cells, and 10 × and 20 × magnifications of myeloid parenchyma cells from WT plants, respectively. Images (**E**–**H**) are 10 × and 20 × magnifications of xylem cells and 10 × and 20 × magnifications of medullary parenchyma cells from *PtSGT1*-1 plants, respectively. Images (**I**–**L**) are 10 × and 20 × magnification of xylem calls and 10 × and 20 × magnifications of myeloid parenchyma cells from *PtSGT4*-1 plants, respectively.

Wood from WT and mutant strains grown for 1 year was sliced by hand. Xylem sections from WT and mutant strains *PtSGT1*-1 and *PtSGT4*-1 were observed using the BX61 microscope (Olympus). Xylem cells from WT plants were arranged tightly and neatly, with small cells (Figure 8). Xylem cells from the mutants *PtSGT1*-1 and *PtSGT4*-1 were loosely arranged and were thinner and larger than those of WT cells. The parenchyma cells in the WT medulla were irregular and disordered in shape, while the parenchyma cells in transgenic *PtSGT1*-1 and *PtSGT4*-1 medulla were polygonal in shape and arranged in an orderly manner.

## 3. Discussion

Cellulose synthesis is a complex process in plant cells, while *SGT* plays an essential role in cellulose synthesis in the cell wall. A previous study found that SG synthesis occurs on the inner surface of the plasma membrane of plant cells and is involved in responses to phytohormones and the initiation reaction of the cellulose synthesis pathway [10]. The mutation of the *Arabidopsis thaliana* co-chaperone *SGT1b* impairs responses to the plant hormones jasmonate, auxin, and gibberellic acid, but not brassinolide and abscisic acid, and that *SGT1b* and its homologue *SGT1a* are involved in maintaining the steady state levels of the F-box proteins COI1 and TIR1, receptors for jasmonate and auxin, respectively [15]. In cotton, two full-length *GhSGT* genes were identified, their distinct biochemical properties were also examined. Using UDP-Glu and β-sitosterol or total crude membrane sterols as substrates, GhSGT1 and GhSGT2 recombinant proteins were detected with different enzymatic activities for SG production [14]. These studies provided a basis for understanding the biological functions of *SGT* gene in plants. In this study, we mutated the *P. tomentosa SGT1* and *SGT4* genes using the CRISPR/Cas9 system, and the results showed that sugar contents of the mutants were significantly different from those of the wild-type. The glucose content in the stem of the *PtSGT1*-1 mutant did not change significantly compared with that in wild-type. Mutation of the plant may have occurred at the 3′-end, which would not alter its function. Further microscopic observation showed that the arrangement and size of parenchyma cells in the xylem and pith of the mutant were altered. It can be inferred from the results that the SGT gene plays a role in the initial reaction of cellulose synthesis, and primarily acts in the xylem region of the stem. The results additionally indicate that SGT could affect the arrangement and size of xylem parenchyma cells. Microscopic observation of the xylem confirmed this result, and the cell wall of the mutant was different from that of wild-type. In addition, we found that the relative expression of *PtSGT3* was greater than *PtSGT1*, *PtSGT2*, and *PtSGT4* in poplar tissues, with the highest expression in stems in a previous study [23]. This suggests that *PtSGT3* may play a crucial role in cellulose synthesis. Therefore, it will be listed as a priority target gene in our future research.

CRISPR/Cas9 has become a powerful tool for functional analysis of desired genes and been widely applied in many plants, including *Arabidopsis*, rice, potato, maize, and tobacco [24,25,26,27,28,29,30,31]. As CRISPR/Cas9 technology advances, researchers are turning their attention to woody plants. Liu et al. [20] assessed the efficiency of mutagenesis using different sgRNAs targeting the *PDS* gene of *P. tomentosa* and found that a mismatch between the sgRNA and target gene led to reduced efficiency of mutagenesis and the possibility of mutagenesis failure. Fan et al. [21] proposed an improved method for infusing Cas9-encoding genes and multiple sgRNAs into plant cells through a single plasmid. CRISPR/Cas9 technology was used in combination with this method to destroy endogenous *PtoPDS* genes in poplar trees. They successfully detected homologous and heterologous PDS mutants, showing that the CRISPR/Cas9 system is an efficient and powerful tool for genomic modification in woody plants. In this study, CRISPR/Cas9 was used to edit the *P. tomentosa SGT* gene. This produced insertion and deletion mutations in the mutant lines, which resulted in changes in the amino acid sequence, predicting that cellulose synthesis may be affected. The changes in glucose metabolism in mutants provided solid evidence. The glucose content in the stem of the *PtSGT4*-1 mutant was higher than in the control, which may be due to the large segment mutation at the 5′-end of the *PtSGT4*-1 plant, resulting in weakened or lost function of the *SGT* gene that could result in continuous accumulation of glucose in the substrate. Due to the high genetic diversity and complex genetic background of *P. tomentosa*, SNPs may exist between different genotypes. Therefore, designing multiple sgRNAs for a target gene and multi-gene editing would be a better strategy for increasing the mutation efficiency in our future work.

## 4. Materials and Methods

### 4.1. Experimental Materials

Tissue-cultured plantlets of *P. tomentosa* (TC1521, female clone) were used for DNA and RNA extraction and genetic transformation. Transformed materials were grown and maintained in the National Forestry Engineering Laboratory of Tree Breeding at Beijing Forestry University, Beijing, China. The plants were cultured in 1/2 Murashige and Skoog solid medium at 25 °C, under 60% relative humidity and a 16 h light/8 h dark photoperiod. The illuminance was 8000–10,000 lx. *Agrobacterium tumefaciens* GV3101 and *Escherichia Coli (E. coli)* Top10 were purchased from Tiangen Biotech (Beijing, China) [32].

### 4.2. Cloning and Sequence Analysis of PtSGT

Total RNA from selected plants was extracted using the SV Total RNA Isolation System kit (Promega, Madison, WI, USA), followed by removal of genomic DNA with RQ1 DNase. Finally, cDNA was synthesized using the Reverse Transcription System according to the manufacturer’s instructions. Based on the CDS of *PtrSGT1* and *PtrSGT4*, DNAMAN v6.0 software was used to design the primers for amplification of *PtSGT1* and *PtSGT4* (Table 3). TargetP 1.1 Server software was used to predict the subcellular localization of the two genes. The PCR reaction amplification system (20 μL) included 10 × PCR Buffer (2.0 μL), 2 mM dNTPs (1.6 μL), forward primers (0.4 μL), reverse primers (0.4 μL), cDNA template (2.0 μL), Taq DNA polymerase (0.2 μL), and ddH_2_O (13.4 μL). The PCR cycle conditions were as follows: 94 °C for 3 min for pre-denaturation, denaturation at 94 °C for 30 s, annealing at 55 °C for 30 s, and extension at 72 °C for 90 s. After 34 cycles, the reactions were extended for 7 min at 72 °C before being preserved at 4 °C.

### 4.3. Construction and Transformation of the PtSGT Vector and Molecular Identification of Transformed P. tomentosa

Using the exon sequence of the *SGT* gene, *PtSGT1-*sgRNA and *PtSGT4-*sgRNA were designed using the online tool CRISPOR (http://crispor.tefor.net/, accessed on 29 October 2021) and assembled into a VK005-14 vector (Beijing Viewsolid Biotechnology Co. Ltd., Beijing, China). The target sgRNA design followed basic principles: the length of the sgRNA target sequence is generally about 20 bp; the 3′ end of the sgRNA target sequence contains GG, and due to its relative specificity, ending with more than 4 T nucleotides is avoided, and the GC content is 40–60%; the number of base matches between the sgRNA sequence and off-target sites should be as small as possible; the PAM (protospacer adjacent motif) NGG motif should be included in the design of the sgRNA target location, but the PAM region should not be included in the design of carrier primers. The integrated vector plasmid VK005-14 was transferred into *E. coli* for single colony PCR detection. After successful detection, positive *E. coli* liquid was extracted for plasmid extraction. The plasmid verified by PCR was introduced into *Agrobacterium tumefaciens* by freeze–thaw method. Explants (2 months old) were obtained from aseptic tissue culture seedlings of the *P. tomentosa* female strain TC1521. Explants were transformed using *Agrobacterium*-mediated leaf disc transformation, with wild-type (WT) plants as controls. The transformation of *P. tomentosa* was performed as described in our previous study [32]. Genomic DNA was extracted from the transformed and WT plants using the Plant Genomic DNA Extraction Kit (Tiangen Biotech, Beijing, China) and served as the PCR template. PCR was performed at 94 °C for 5 min, followed by 34 cycles of amplification (94 °C for 30 s, 58 °C for 30 s, and 72 °C for 1 min), with a final extension at 72 °C for 5 min. The PCR products were analyzed using 1.5% agarose gel electrophoresis [32]. Primers used for mutation sequencing analysis are listed in Table 1. The thermal cycles followed the same conditions mentioned above.

### 4.4. Sugar Concentration Measurements in Transgenic Line Tissues

The root, stem, and leaf tissues of mutant and wild-type (TC1521) *P. tomentosa* were collected. Three duplicate samples were taken from each tissue. Sugars were extracted by drying fresh tissue in an oven and grinding the sample. Samples (~10 mg) were added to 0.8 mL of 80% ethanol solution, placed in a water bath for 40 min at 80 °C, and centrifuged (10,000 rpm, 1 min) before collecting the supernatant. These steps were repeated before the addition of ~2 mg activated carbon to the supernatant. The supernatant was decolorized for 30 min at 80 °C and centrifuged (10,000 rpm, 1 min) before adding 80% ethanol solution for a final volume of 2 mL [32].

Sucrose content was measured in the transgenic lines by adding NaOH (0.1 mL; 2 mol/L) to the sugar extract (0.2 mL) and boiling the reaction mixture in water for 5 min. HCL (1.4 mL; 30%) and resorcinol (0.4 mL; 0.1%) were added to the reaction and the mixture was shaken at 80 °C for 5 min. The absorbance of the reaction was measured at 480 nm to determine the sugar concentration. Fructose content was analyzed by adding HCl (0.8 mL; 30%) and resorcinol (0.8 mL; 0.1%) to a 2 mL tube containing 0.4 mL of sugar extract. Following incubation at 80 °C for 10 min, the absorbance was measured at 520 nm to determine the fructose concentration. The glucose content was analyzed by incubating 100 mL enzyme preparations (10 mg horseradish peroxidase, 10 mg o-dianisidine-HCl, 0.1 mL glucose oxidase (dissolved with 1 mg 50,000 U glucose peroxidase in 0.1 mol/L glacial acetic acid, pH 5.5) in a constant volume to 100 mL) at 30 °C for 5 min, then 0.5 mL was added to 0.25 mL sugar extract, the mixture was shaken, and we then allowed it to sit for 5 min. Finally, H_2_SO_4_ (1 mL; 10 mol/L) was added to stop the reaction. The glucose concentration was determined by measuring the absorbance of the reaction mixture at 520 nm. The calculation methods followed were the same as those seen in a previous publication [32].

### 4.5. Sections of Xylem from Transgenic Plants

Wood from WT and mutant strains grown for 1 year was sliced by hand. Xylem sections of WT and mutant strains *PtSGT1*-1 and *PtSGT4*-1 were observed and photographs were taken using a BX61 microscope (Olympus, Tokyo, Japan). The sections were placed in FAA (Formaldehyde-acetic acid–ethanol) solution (38% formaldehyde 5 mL, glacial acetic acid 5 mL, 70% alcohol 90 mL) for fixation for 15–60 min. The slices were transferred to a small Petri dish and dehydrated with 50, 60, and 70% ethanol for 5 min each. Then they were dyed for at least 30 min in 1% safranin dissolved in 70% ethanol. The stained slices were dehydrated with 70, 80, 90, and 95% ethanol for 5 min each. The sections were stained with 95% ethanol containing 1% fast green for 30 s. The slices were placed in 95% ethanol for color separation and dehydrated with anhydrous ethanol for 5 min. The sections were transferred to a 1:1 anhydrous ethanol: xylene mixture for 4 min, and then transferred into pure xylene for 5 min to make the tissue sections transparent. Then, the section was placed on a slide with a drop of water, the cover slide was gently lowered, and the excess water was absorbed with filter paper, and then observed with a BX61 microscope.

## 5. Conclusions

In this study, we produced mutated *P. tomentosa* lines of *PtSGT1* and *PtSGT4* using CRISPR/Cas9. The results showed that there were some changes in sugar content and xylem structure in transgenic plants compared with wild-type. It is speculated that sucrose metabolism pathway is affected, suggesting important roles for *PtSGT1* and *PtSGT4* in cellulose synthesis. This work promotes our understanding of cellulose synthesis in the cell walls of woody plants.

## Figures and Tables

**Table 1:** The sgRNA target sequences.

Gene	Name of Plasmid	sgRNA Target Sequence (5′→ 3′)
*PtSGT1*	VK005-14-PtSGT1	CCCATACCTGTTGATGAGTTC
*PtSGT4*	VK005-14-PtSGT4	TAGATCCAAGACGGAGAACAGGG

**Table 2:** List of universal detection primers for plasmid vectors.

Primer	Sequence (5′→3′)
Universal detection primer F	TCTTCAAAGTCCCACATCGC (Source: VK005-14)
Universal detection primer R	ACGCTAAGGGAATGCTTGTAT (Source: VK005-14)

**Table 3:** PCR primer sequences for *PtSGT1* and *PtSGT4* amplification and mutation sequencing analysis.

Primer	Sequence (5′→3′)	Purpose
*PtSGT1*F	ATGGCGGAGTCGCAGC	Used for *PtSGT1* and *PtSGT4* amplification
*PtSGT1*R	TCAGGGACAACCAAAACATTTACT	
*PtSGT4*F	ATGGTTAATAATAGATTTGATCATC	
*PtSGT4*R	TTATGAACAGGATAGGCAG	
*PtSGT1*Ft	TATGCTTGTAATACCGATGC	Used for mutation sequencing analysis
*PtSGT1*Rt	TGAGTAGCCAGTCTAACACGAT	
*PtSGT4*Ft	CAGAAGACAAAGTCAGGGAA	
*PtSGT4*Rt	TCTAACAACTCCGAAGCAAC	

## Data Availability

The data presented in this study are openly available in the Sequence Read Archive of NCBI, the accession number of *PtSGT1* is MG904686, and the accession number of *PtSGT3* is MG904687. The remaining data are provided in the Supplementary Files.

## Supplementary Materials

The following are available online at https://www.mdpi.com/article/10.3390/ijms222413200/s1.
