# Supplementary material for: Investigation of PtSGT1 and PtSGT4 Function in Cellulose Biosynthesis in Populus tomentosa Using CRISPR/Cas9 Technology
Source: Int J Mol Sci. 2021 Dec 7;22(24):13200. doi: 10.3390/ijms222413200 (PMC8704405; doi:10.3390/ijms222413200)
Supplement: Supplementary file 1 [file ijms-22-13200-s001.zip › tables.pdf]

Supplementary Table S1. List of the number of indel events.

Mutant Strain	Indel			
	−1	+1	Conversion	No Change
PtSGT1	1	0	2	2
PtSGT4	0	1	0	0

Supplementary Table S2.

Gene Name	Gene ID	Gene Size	Chromosome Location	Isoelectric Point	Transmembrane
PtSGT1	MG904686	1851	Chr.14	6.02	inexistence
PtSGT2	unpublished	2136	Chr.2	6.17	inexistence
PtSGT3	MG904687	1911	Chr.2	5.79	inexistence
PtSGT4	unpublished	1935	Chr.5	6.05	inexistence

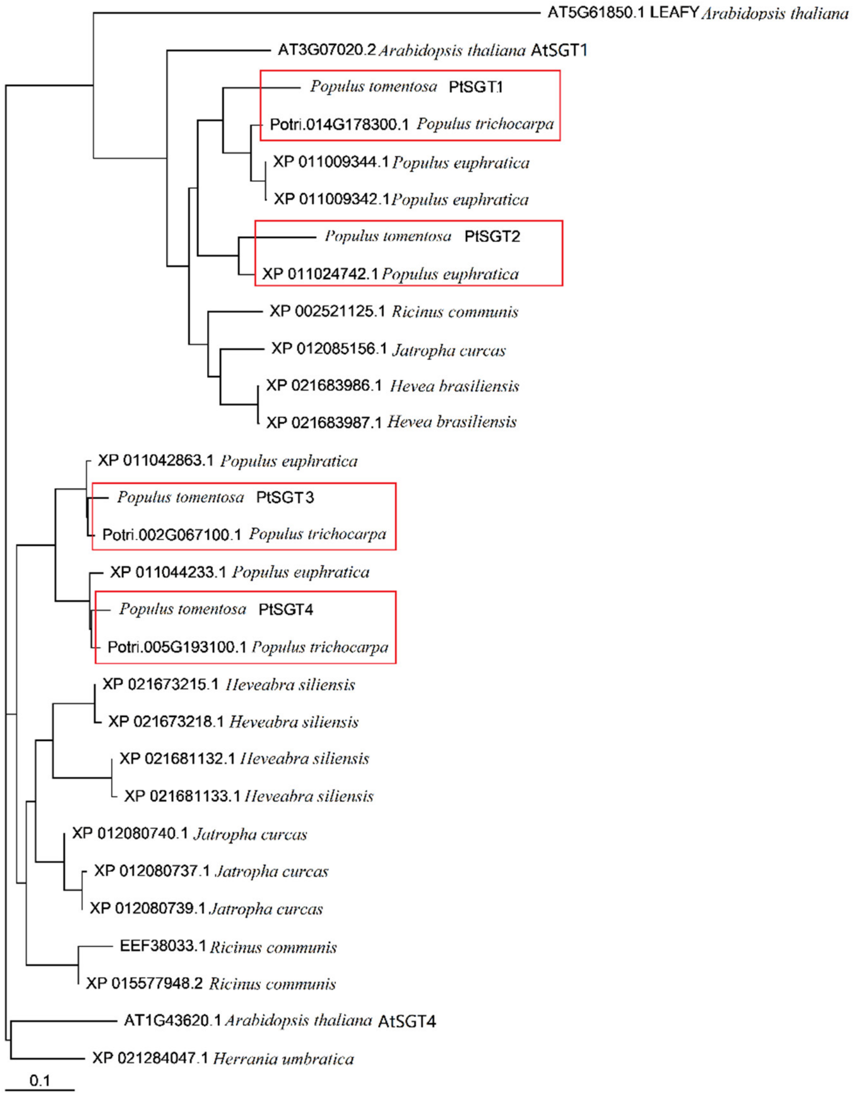

Supplementary Figure S1. Phylogenetic tree of PtSGT1、PtSGT2、PtSGT3 and PtSGT4 with other species.
